# Ivermectin induces apoptosis of esophageal squamous cell carcinoma via mitochondrial pathway

**DOI:** 10.1186/s12885-021-09021-x

**Published:** 2021-12-07

**Authors:** Nana Xu, Mengmeng Lu, Jiaxin Wang, Yujia Li, Xiaotian Yang, Xiajie Wei, Jiaoyang Si, Jingru Han, Xiaojuan Yao, Juanmei Zhang, Junqi Liu, Yanming Li, Hushan Yang, Dengke Bao

**Affiliations:** 1 Laboratory of Cancer Biomarkers and Liquid Biopsy, School of Pharmacy, Huaihe Hospital, Henan University, Kaifeng, 475004 Henan China; 2 Department of Radiation Oncology, the First Affiliated Hospital of Zhengzhou University, Zhengzhou, 450052 Henan China; 3 Department of Cardiology, Huaihe Hospital, Henan University, Kaifeng, 475000 Henan China; 4 Department of Medical Oncology, Sidney Kimmel Cancer Center, Thomas Jefferson University, Philadelphia, PA 19107 USA

**Keywords:** ESCC, Ivermectin, Apoptosis, Mitochondrial, NF-κB

## Abstract

**Background:**

Esophageal squamous cell carcinoma (ESCC) is the most predominant primary malignant tumor among worldwide, especially in China. To date, the successful treatment remains a mainly clinical challenge, it is imperative to develop successful therapeutic agents.

**Methods:**

The anti-proliferative effect of ivermectin on ESCC is investigated in cell model and in nude mice model. Cell apoptosis was assessed using flow cytometry, TUNEL assay and western blotting. Mitochondrial dysfunction was determined by reactive oxygen species accumulation, mitochondrial membrane potential and ATP levels.

**Results:**

Our results determined that ivermectin significantly inhibited the proliferation of ESCC cells *in vitro* and *in vivo*. Furthermore, we found that ivermectin markedly mediated mitochondrial dysfunction and induced apoptosis of ESCC cells, which indicated the anti-proliferative effect of ivermectin on ESCC cells was implicated in mitochondrial apoptotic pathway. Mechanistically, ivermectin significantly triggered ROS accumulation and inhibited the activation of NF-κB signaling pathway and increased the ratio of Bax/Bcl-2.

**Conclusions:**

These finding indicated that ivermectin has significant anti-tumour potential for ESSC and may be a potential therapeutic candidate against ESCC.

**Supplementary Information:**

The online version contains supplementary material available at 10.1186/s12885-021-09021-x.

## Background

Esophageal squamous cell carcinoma (ESCC) is one of the most common and fatal malignancies in China [1, 2], which is considered as an aggressive cancer due to its poor prognosis and high mortality rate with a 5-year survival rate of only about 20% [3–5]. Since most patients diagnosed with ESCC were found to have locally advanced or metastatic disease and thus are not candidates for radical surgical resection, these patients are treated with radiotherapy and chemotherapy [6, 7]. However, few candidate drugs that have long-term benefits for ESCC therapy and the resistance of ESCC cells to chemotherapy, which suggests that it is urgent to identify novel therapeutic alternatives or agents to improve systemic therapy for ESCC patients.

Ivermectin is a polycyclic lactone pesticide produced by streptomyces avermitilis and is found to be a broad-spectrum effect against parasites [8, 9]. Moreover, some studies had revealed the anti-proliferative potential of ivermectin in colon cancer, ovarian cancer, melanoma and leukemia by inducing cell apoptosis and cell cycle arrest, activating necrosis pathways and suppressing tumor initiation and malignant transformation [10–12]. Previous study demonstrated that the growth of breast cancer cells can be inhibited by ivermectin by inducing autophagy [13]. Ivermectin can also inhibit the cells growth of human ovarian cancer through blocking the oncogenic kinase PAK1 [14]. However, the effect and molecular mechanism of ivermectin on ESCC growth has not been clearly determined.

Inhibiting proliferation and increasing the sensitivity of cancer cells to undergo apoptosis are considered important properties in developing novel chemotherapeutic agents [15]. Mitochondria are important mediators of tumorigenesis and targeting mitochondrial-associated apoptotic pathways is a potential therapeutic strategy for cancer [16]. In this study, we first evaluated the anti-proliferative effect of ivermectin on ESCC cells *in vitro* and *in vivo*. Our results shown that ivermectin inhibit ESCC cells growth and induce ESCC cells apoptosis through mitochondria-dependent ROS production. Further, we demonstrate that ivermectin induced mitochondrial dysfunction and ROS accumulation of ESCC cells, which subsequently inhibited the activation of NF-κB signaling pathway and induced ESCC cells apoptosis. These findings demonstrated that ivermectin may be a potential candidate drug for ESCC therapy.

## Methods

### Cell culture and treatment

Esophageal squamous carcinoma cell lines and Normal esophageal epithelial cells, KYSE-70, KYSE-30 and NE-3 were purchased from Nanjing Kebai Biotechnology Co., ltd. The cells were cultured in RPMI-1640 (10040-CVR, Corning) medium containing 10% fetal bovine serum (04-001-1Acs, BI) in a 37°C 5% CO_2_ incubator, and cells were grown to 65%-75% confluence and treated under various conditions as indicated.

### Cell viability, proliferation and lactate dehydrogenase (LDH) leakage assays

ESCC cells and NE-3 were seeded in 96-well plates (5×10^5^ cells/well or 1×10^5^ cells/well) for 24 h, and treated with ivermectin (S1351, Selleck Chemicals) at serially diluted concentrations of 2.5, 5, 10, 15, 20 μM on NE-3 and KYSE-70, 2, 4, 6, 8, 10 μM on KYSE-30 for 48 h. We found that the half maximal-inhibitory concentration (IC_50_) of ivermectin for KYSE-70 and KYSE-30 cells was close to 10 μM and 6 μM, respectively. Thus, 10 μM and 6 μM ivermectin were used for further experiments to evaluate the anti-proliferative effect on ESCC cells. MTT assay was performed to assess the viability of ESCC as described previously [17]. ESCC cells were seeded at a density of 2000 cells/well or 500 cells/well in 6-well plates, incubated for 24 h, and then exposed to ivermectin for 10-14 days. Next, the medium was removed and cells were fixed with 4% paraformaldehyde for 15 min, washed three times with PBS and incubated with 0.5% crystal violet solution (C8470, Soiarbio) for another 5 min. The colonies were counted and assays from three independent experiments. EdU (C10310, Ribobio) incorporation assay were used to evaluate the proliferation of cells according to the manufacturer’s instructions. Then results were analyzed with a fluorescence microscope (Olympus Corporation U-LH100HG). LDH leakage was measured using a colorimetric LDH assay kit (C0017, Beyotime) following the manufacturer’s recommended protocol. For relative quantification, the value of absorbance in each group was normalized to the control group. Cell cycle of ESCC cells after treatment with ivermectin is analyzed using Cell cycle kit (C6031, US Everbright) following manufacturer’s instructions and determined by flow cytometry using CytoFLEX (Beckman Coulter).

### Cell apoptosis assay

Cell apoptosis was assessed using the Annexin-V-FITC apoptosis detection kit (C6031, US Everbright). ESCC cells were seeded in 6-well plates for 24 h, and treated with ivermectin at 5, 10, 15 μM on KYSE-70 and 4, 6, 8 μM on KYSE-30 for 48 h, the apoptosis was assessed following the manufacturer’s protocol and analyzed by a flow cytometry (FACSVerse, BD), and the data were analyzed with Flowjo VX10 Software (VX10, USA). Cell apoptosis was also detected by using the TUNEL assay kit (T6013, UE) and Hoechst 33342 (H4047, UE) staining following the manufacturer’s protocol. The cellular nuclei were stained with the TUNEL reaction overnight at 4°C and fixed with 4% paraformaldehyde for 5min. After washing three times using PBS, the cells were incubated with Hoechst 33342 for 10 min. The TUNEL-positive cells (green) and Hoechst 33342 (blue) staining patterns were detected by fluorescence microscope (Olympus Corporation U-LH100HG).

### Detection of reactive oxygen species, mitochondrial membrane potential (MMP) and ATP production

The cells were planted in 6-well plates and cultured overnight, exposed to ivermectin at 37°C for 48 h. Cellular reactive oxygen species (ROS) levels were detected by the fluorescent probe DCFH-DA following the manufacturer’s protocols (S0033S, Beyotime) [18]. Briefly, ESCC cells suspension were incubated with 10 μM DCFH-DA for 20min at 37°C and analyzed by a flow cytometry (FACSVerse, BD), and the data were analyzed with Flowjo VX10 Software (VX10, USA). The alteration of cellular MMP was evaluated using JC-1 kit (C2006, Beyotime). Cells were adjusted to a density of 5 × 10^5^/ml and stained with 2 μg/ml JC-1 dye for 20 min at 37°C, the results were observed by a fluorescence microscope (Olympus Corporation U-LH100HG). The total ATP levels were determined using the Cell Titer-Glo Luminescent assay (FF2000, Promega) according to the manufacturer’s instructions.

### Subcutaneous xenograft models

5-week-old female Balb/c nude mice (Beijing Charles River Laboratories, Beijing, China) were randomly divided into indicated groups (n=5). 5 × 10^6^ ESCC cells in 20 μL serum free RPMI-1640 was injected into the right flanks to establish the subcutaneous xenograft nude mice model. Tumor size was measured every two days, and the tumor volume was calculated according to (Width^2^ × Length)/2. When the tumor volumes reached 100 mm^3^, mice were randomized into two groups receiving 0.1 mL of saline or 25 mg/kg ivermectin per nude mice, respectively. The nude mice were monitored twice a week for palpable tumor formation. Then, all mice were euthanized by intravenous with 100 mg/kg sodium pentobarbital (P3761, Sigma) and the tumors were harvested and weighted. All experiments in this study were performed in accordance with relevant guidelines and regulations and were approved by Ethics Committee of Henan University. Animal experiments were carried out in compliance with the ARRIVE guidelines and was approved by the Institutional Animal Care and Use Committee of Henan University (HUSOM-2019-031).

### qPCR, Western blot, IHC

Total RNA extraction, complementary DNA synthesis, and qPCR were performed as previously described [19]. Primer sequences of qPCR were provided in Supplementary Table 2. Preparation of whole-cell protein lysates and western blotting analysis were performed as we described before [17, 20]. Primary antibodies used in this study included anti-P65 (8242T; D14E12; Cell Signaling), anti-pP65 (3033T; 93H1; Cell Signaling), anti-IκBα (4814T; L35A5; Cell Signaling), anti-pIκBα (2859T; 14D4; Cell Signaling), anti-PARP-1 ( sc-56197; 194C1439; Santa Cruz Biotechnology), anti-cleaved PARP-1 (sc-56196; 5A5; Santa Cruz Biotechnology), anti-Bcl-2 (12789-1-AP; Polyclonal Antibody), anti-Bax(50599-2-lg; Polyclonal Antibody), anti-Cleaved-caspase-3 (CY5031; Abways), and anti-Cleaved-caspase-9 (CY5682; Abways). The IHC staining (D601037, Sangon Biotech) was detected by using immunohistochemistry kit according to the manufacturer’s instructions. The cleaved caspase 3 (CY5031; Abways), ROS1 (ab189925; EPMGHR2; Abcam), p-p65 (3033T; Cell Signaling) and Ki-67 (CY5542; Abways)-positive staining was detected using a microscope (Nikon, E100) and quantification using the ImageJ software (NIH, Bethesda, MD, USA). Details of reagents used in this study were provided in Supplementary Table 1.

### Statistical treatment

The results were expressed as the mean ± SD. Mean values were calculated from data obtain from experiments performed in triplicate. The differences between the experimental and control groups were compared using one-way ANOVA followed by Dunnett’s multiple comparisons test. Statistical software SPSS20.0 was used in data processing and for analyzing the significance between groups with the t-test. *p* < 0.05 was considered statistically significant.

## Results

### Ivermectin inhibits proliferation of ESCC cells in vitro

**Fig. 1:**
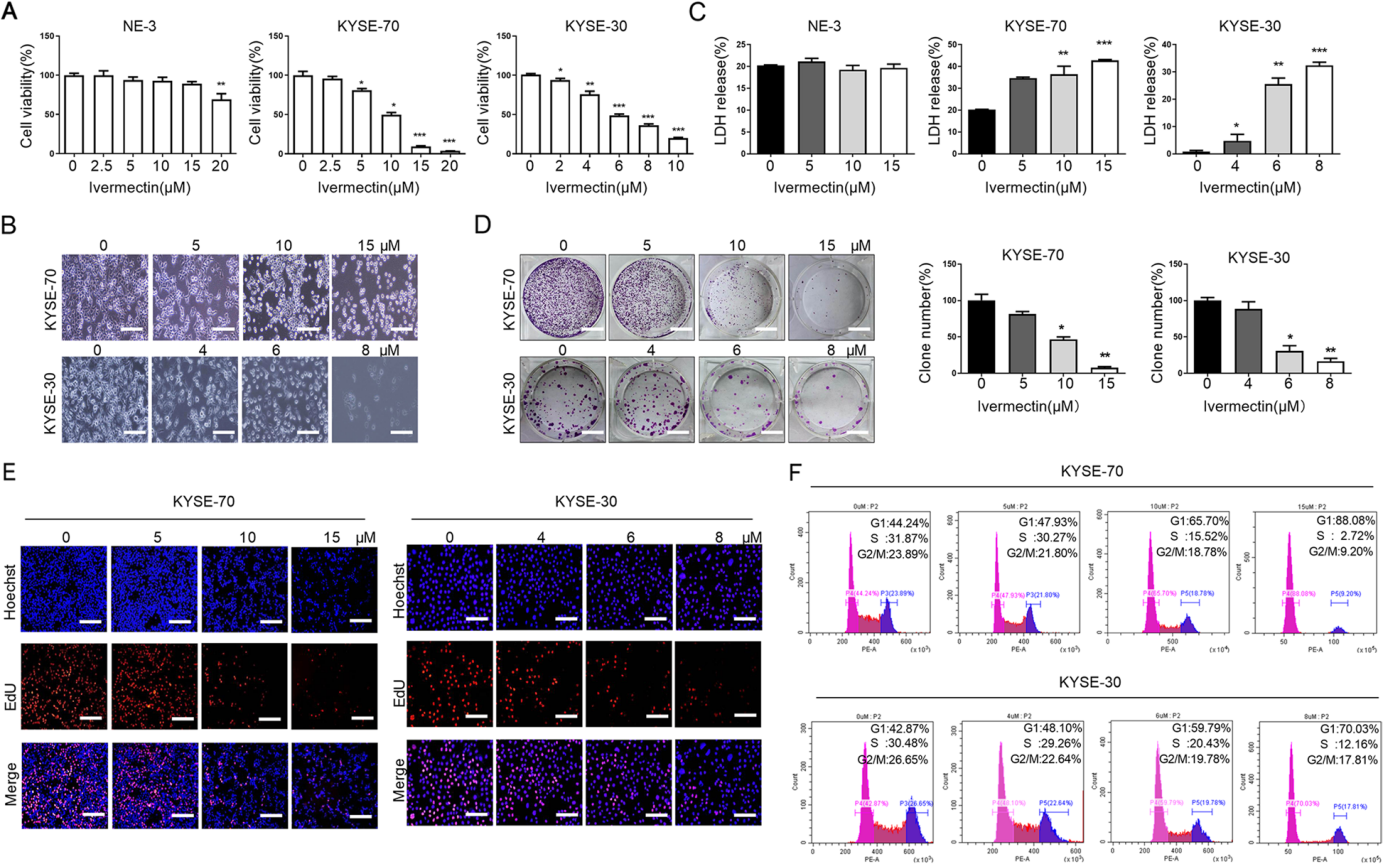
Ivermectin inhibits proliferation of ESCC cells *in vitro*. **A**. Viability of cells was assessed by MTT assay. **B**. The morphological changes of ESCC cells were observed by phase contrast microscopy at 200× magnification, Scale bar = 50 μm; The anti-proliferative effect of ivermectin on ESCC cells was determined by LDH assay (**C**); colony formation (**D**), Scale bar = 10 mm; and EdU incorporation assays (**E**), Scale bar = 50 μm; **F**. Cell cycle of ESCC cells after treatment with was performed by flow cytometry. Values represent mean ± S.E.M. of 3 independent experiments. ^*^P < 0.05, ^**^P < 0.01; ^***^P < 0.001.

To investigate the anti-proliferative effect of ivermectin on ESCC cells, the MTT assay was conducted to assess the growth of ESCC cells after incubated with ivermectin for 48h. As shown in Fig. 1A, ivermectin markedly decreased the cell viability of ESCC cells with a dose-dependent manner. Moreover, the half maximal-inhibitory concentration (IC_50_) of ivermectin for KYSE-70 and KYSE-30 cells was close to 10 μM and 6 μM, respectively. We also found that the typical morphological characteristic of ESCC cells was changed after ivermectin treatment for 48h. The morphology of ESCC cells with ivermectin treatment became irregular, wrinkled or even broken and smaller and roundness than the control group (Fig. 1B). Lower concentrations of ivermectin (2.5-15 μM) had no cytotoxic effect in NE-3 cells, while 20 μM ivermectin slightly inhibited viability of NE-3 cells viability (Fig. 1A). We next performed another cytotoxicity evaluation in ESCC cells after ivermectin treatment using the LDH cytotoxicity assay kit after ivermectin treatment. As shown in Fig. 1C, treatment with ivermectin significantly increased LDH release in the medium, which was consistent with MTT assay. The anti-proliferative effect of ivermectin on ESCC cells was determined by colony formation and EdU incorporation assays. As shown in Fig. 1D, the proliferative potential and colony formation ability of ESCC cells were remarkably suppressed after ivermectin treatment. EdU incorporation was remarkably reduced in ivermectin-treated ESCC cells compared to controls (Fig. 1E). Cell cycle analysis performed using flow cytometry indicated that ivermectin significantly decrease the population of cells in S and G2/M phase and increase the population of G1 with a concentration-dependent manner (Fig. 1F). Collectively, these results demonstrate that ivermectin inhibits the proliferation of ESCC cells in *vitro*.

### Ivermectin promotes apoptosis of ESCC cells

**Fig. 2:**
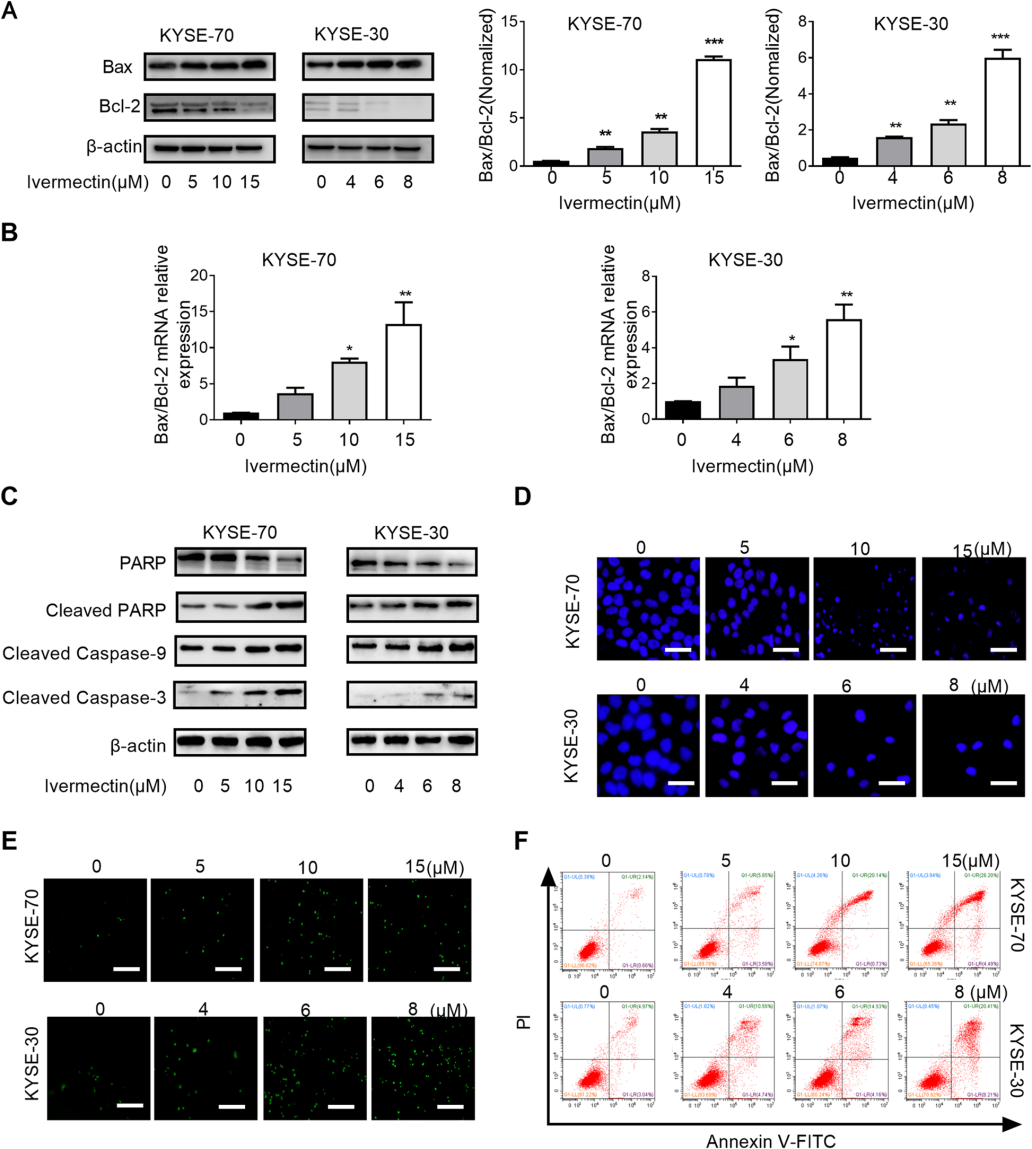
Ivermectin promotes apoptosis of ESCC cells. Western blotting (**A**) and qRT-PCR (**B**) analysis for the expression levels of Bax and Bcl-2 after ivermectin treatment; **C**. The expression of caspase cascade (cleaved-caspase 9, cleaved-caspase 3, PARP, cleaved PARP) were analyzed by western blotting; **D**. Cells were observed by fluorescence microscopy after staining with Hoechst 33342, Scale bar = 100 μm; **E**. Ivermectin-induced ESCC cells apoptosis was determined by TUNEL assay (200×), Scale bar = 50 μm; **F**. Ivermectin-induced ESCC cells apoptosis was determined by PI/Annexin-V assay using flow cytometry. ^*^P < 0.05, ^**^P < 0.01; ^***^P < 0.001.

Apoptosis is a major form of cell death for cancer cells induced by chemotherapeutic agents and implicated in the cytotoxic mechanisms of various classes of chemotherapy [21]. Therefore, we first simultaneously evaluated the expression level of Bax and Bcl-2, two apoptosis related proteins, and analyzed the ratio of Bax/Bcl-2, simultaneously. As shown in Fig. 2A and B, the expression level of pro-apoptotic factor Bax was significantly increased after ivermectin treatment, while the expression of anti-apoptotic factor Bcl-2 was markedly decreased when compared with control group. Thus, the ratio of Bax/Bcl-2 was significantly increased in ivermectin-treated ESCC cells. The activation of the caspase cascade is well known intrinsic cell apoptotic pathway via mitochondria. The expression levels of cleaved-caspase-9, cleaved-caspase-3 and caspase downstream effectors (PARP) were estimated by western blot analysis. As shown in Fig. 2C, after ivermectin treatment, the levels of cleaved-caspase 9, cleaved-caspase 3 and cleaved-PARP protein were remarkably increased. We further detected whether ivermectin induced apoptosis in the ESCC cells by Hoechst 33342 staining assays and flow cytometry analysis using Annexin V-FITC/PI kit. Hoechst 33342 staining assays showed that the nuclear morphology of ESCC cells exposure to ivermectin were condensed, fragmented and crescent shaped, while the control cells had a round, bright and regular morphology (Fig. 2D). Using TUNEL and Annexin-V FITC/PI assays by flow cytometry, we showed that the percentage of apoptotic ESCC cells was significantly increased in the ivermectin group compared to control (Fig. 2E and F). Taken together, these data suggested that the inhibitory effect of ivermectin on ESCC cells was associated with cell apoptosis.

### Ivermectin mediates mitochondrial dysfunction of ESCC cells

**Fig. 3:**
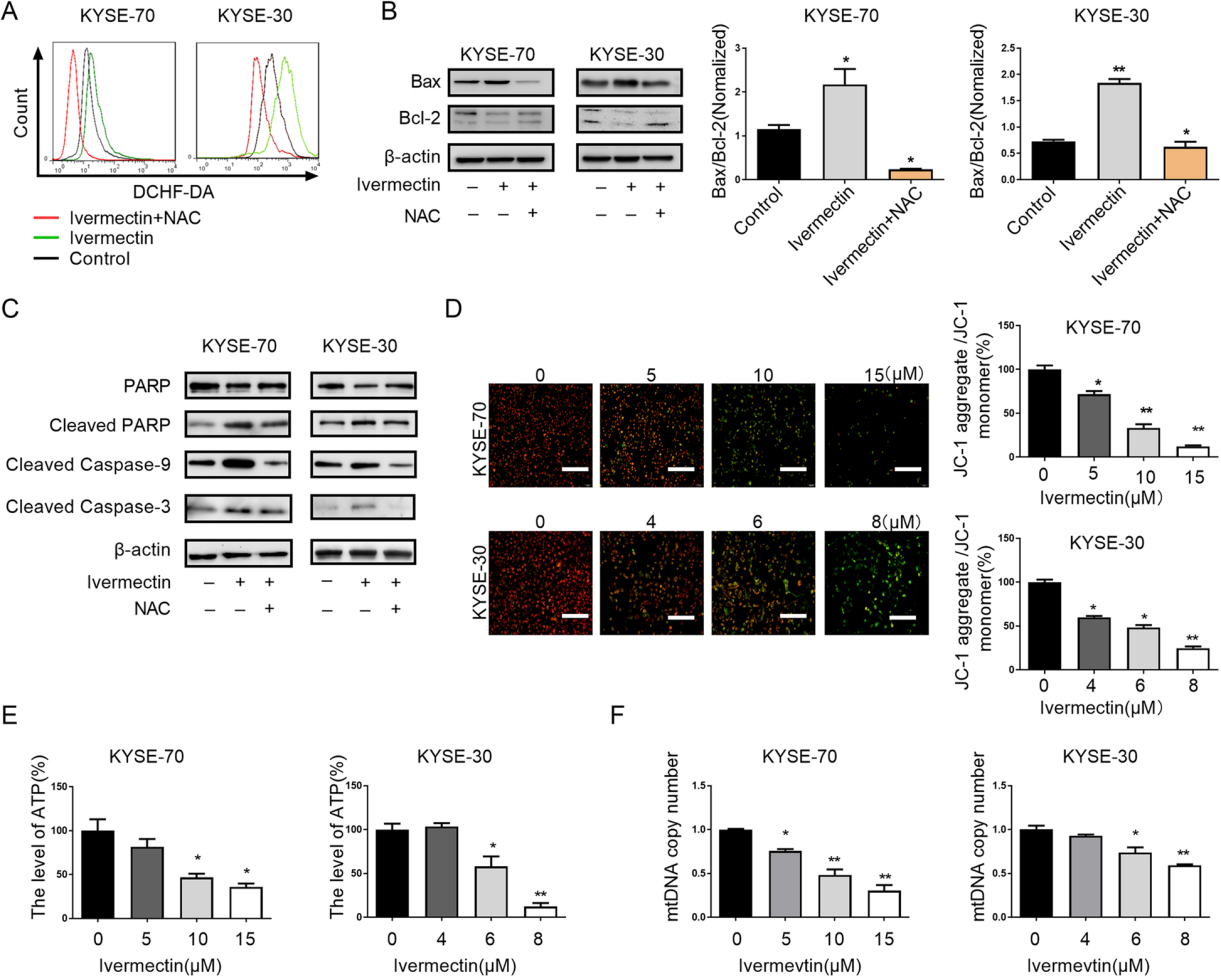
Ivermectin mediates mitochondrial dysfunction of ESCC cells. **A**. Intracellular ROS levels were investigated by DCFH-DA fluorescence in ESCC cells treatment with indicated; **B**. The expression levels of Bax and Bcl-2 were determined by western blot analysis; **C**. The expression of caspase cascade (cleaved-caspase 9, cleaved-caspase 3, PARP, cleaved PARP) were analyzed by western blotting; **D**. Mitochondrial membrane potential was observed by fluorescence microscope at 200 × magnifications, Scale bar = 50 μm; **E**. ATP production of ESCC cells after treated with ivermectin was detected; **F**. The mitochondrial DNA copy number in ESCC cells treatment with invermectin was evaluated using qRT-PCR. ^*^P < 0.05, ^**^P < 0.01.

Intracellular ROS, which are predominantly derived from the mitochondria, are known to induce oxidative damage and ultimately trigger a series of mitochondrial associated events, including apoptosis. Consistent with previous research, we found that ivermectin induced intracellular ROS accumulation in ESCC cells [12]. To determine whether the ROS accumulation was functionally important for ivermectin-induced apoptosis, we added the free radical scavenger N-acetyl-L-cysteine (NAC) to ivermectin-treated cells. NAC markedly eliminated intracellular ROS induced by ivermectin (Fig. 3A) and abrogated ivermectin-induced the increase of Bax/Bcl-2 ratio (Fig. 3B). Consistent with this data, the expression of cleaved caspase 9, cleaved caspase 3 and cleaved PARP proteins in ESCC cells were significantly decreased after exposure to ivermectin and NAC, when compared with ivermectin alone (Fig. 3C).

It had been demonstrated that mitochondria-dependent apoptotic pathway play a vital role in drug-induced apoptosis [22]. To determine whether ivermectin induced ESCC cells apoptosis though a mitochondria-dependent pathway, we examined the mitochondrial function by mitochondrial membrane potential, mitochondrial DNA contents change and ATP production. As shown in Fig. 3D, we found that more ESCC cells with green or orange fluorescence, which indicated that mitochondrial membrane potential was decreased after ivermectin treatment. Ivermectin significantly decreased ATP production in ESCC cells with a time-dependent manner (Fig. 3E). To further verify the hypothesis, the mitochondrial DNA contents change in ESCC cells were detected after ivermectin treatment. Simultaneity, our results indicated that mitochondrial DNA contents were significantly reduced in ESCC cells treatment with ivermectin (Fig. 3F). These results indicated that ivermectin-induced ESCC cells apoptosis was associated with mitochondrial dysfunction.

### Ivermectin induces apoptosis of ESCC cells through NF-κB pathway

**Fig. 4:**
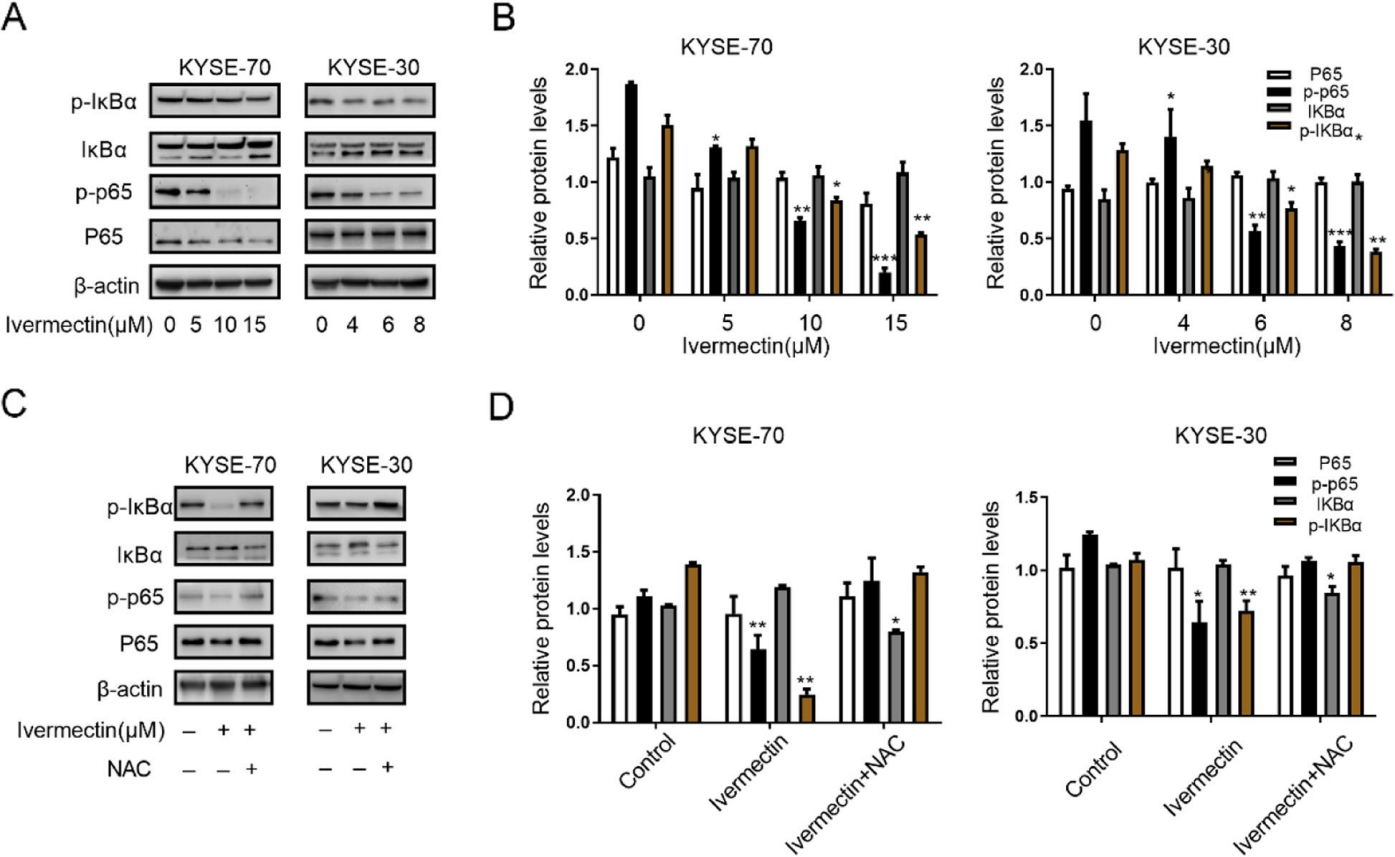
Ivermectin induces apoptosis of ESCC cells through NF-κB pathway. **A** and **C**. Western blotting analysis for the expression levels of p65, p-p65, p-IκBα and IκBα in ESCC cells after ivermectin treatment; **B** and **D**. Quantified for the protein expression levels of (**A**) and (**C**). ^*^P < 0.05, ^**^P < 0.01; ^***^P < 0.001.

The NF-κB signaling pathway is involved in ESCC carcinogenesis and progression and is hyperactivated in ESCC cells and promotes cell survival. To evaluate whether NF-κB signaling pathway was implicated in ivermectin-mediated anti-tumor properties of ESCC, the expression of related signaling molecules in NF-κB pathway were measured. We observed that ivermectin significantly decreased the phosphorylation and nuclear translocation of NF-κB p65 protein in a concentration-dependent manner (Fig. 4A and B). Subsequently, we also found that the phosphorylation of IκBα, an upstream regulatory molecule of NF-κB pathway, was inhibited in ESCC cells after ivermectin treatment (Fig. 4A and B). Consistent with previous study, eliminating intracellular ROS by NAC markedly abrogated ivermectin-induced inactivation of NF-κB pathway, observed through the increased phosphorylation of IκBα and NF-κB p65 protein (Fig. 4C and D). Taken together, these results indicated that ivermectin induces apoptosis of ESCC cells through NF-κB pathway.

### Ivermectin suppressed xenograft growth of ESCC cells in vivo

**Fig. 5:**
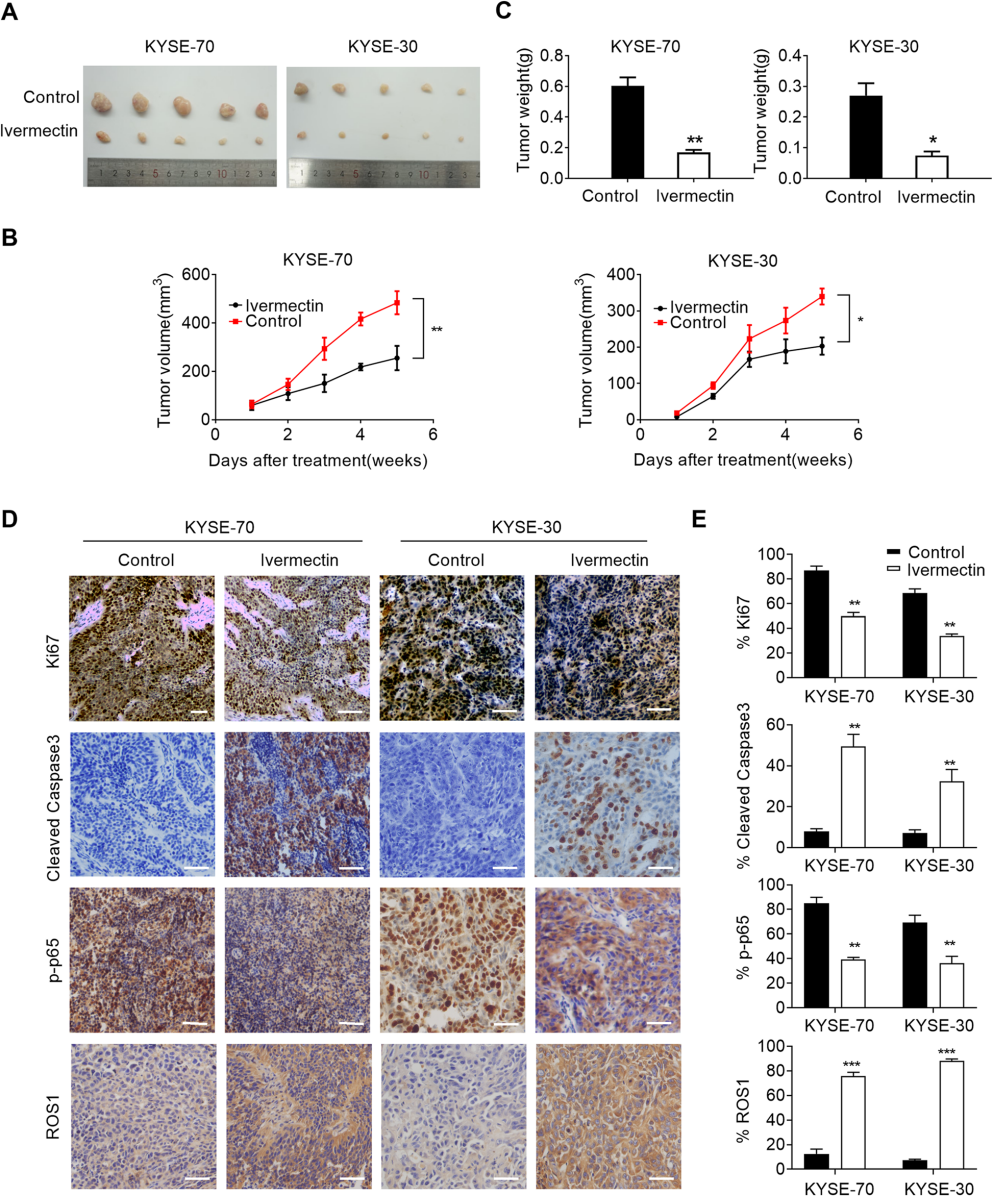
Ivermectin suppressed xenograft growth of ESCC cells *in vivo*. **A**, **B** and **C**. Tumor growth curves and weight of subcutaneous xenograft tumor model developed from ESCC cells treatment with ivermectin as indicated (n=5); **D** and **E**. Representative immunohistochemistry images of Ki67, ROS1, p-p65 and cleaved-caspase 3 in xenograft tumor developed from KYSE-70 and KYSE-30 cells treatment with ivermectin as indicated. Scale bar: 300μm. ^*^P < 0.05, ^**^P < 0.01; ^***^P < 0.001.

To evaluate the anti-proliferative effects of ivermectin on ESCC growth *in vivo*, xenograft nude mice model was established by subcutaneously injecting ESCC cells. We observed that the growth potential of ESCC cells-derived subcutaneous tumor was remarkably attenuated by treatment with ivermectin. The size of xenografts tumor treated with ivermectin was significantly smaller than control saline groups (Fig. 5A) and the proliferation rate in ivermectin treatment group was slower (Fig. 5B). Tumor weight was also markedly reduced in ivermectin-treated mice when compared to control group (Fig. 5C). Furthermore, the percentage of Ki67-positive cells in xenografts tumor by injection of ivermectin was also notably attenuated (Fig. 5D and E). The p-p65 expression in nuclear was significantly decreased in xenografts tumor cells by injection of ivermectin (Fig. 5D and E). However, the expression of ROS1 and cleaved caspase 3 were remarkable increased in ivermectin treatment group (Fig. 5D and E). Taken together, these data suggest that ivermectin inhibited the growth of ESCC cells both *in vivo*.

## Discussion

**Fig. 6:**
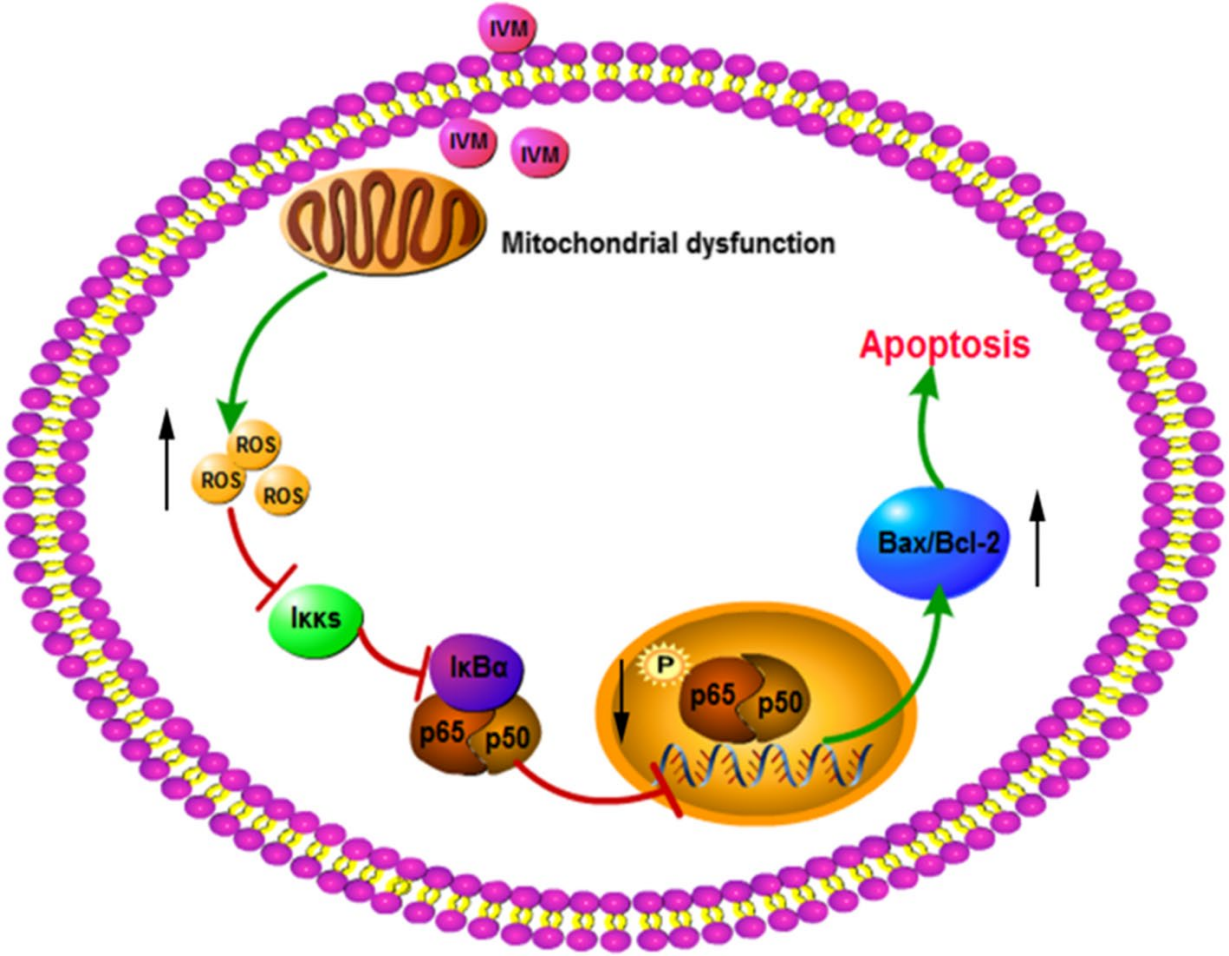
Schematic depicting the anti-proliferative potential of ivermectin on ESCC cells.

In this study, the anti-proliferative potential of ivermectin on ESCC cells was evaluated. Our results demonstrated that ivermectin could inhibit the proliferation of ESCC cells *in vivo* and *in vitro*. Furthermore, we found that ivermectin induced apoptosis of ESCC cells, which was implicated with mitochondrial pathway. In addition, the apoptosis induced by ivermectin triggered by mitochondrial dysfunction-derived ROS through NF-κB signaling pathway (Fig. 6).

Recent studies demonstrated that antiparasitic drugs can inhibit the growth of multiple cancers, such as melanoma, ovarian cancer, breast cancer, glioblastoma [11, 13, 23, 24]. Here, we found that treatment with ivermectin conspicuously suppressed the proliferation of ESCC cells by MTT, colony formation and EdU incorporation assay and were demonstrated *in vivo* of xenografts tumor. All control xenografts displayed stronger Ki67 staining than that of ivermectin-treated mice. Taken together, these data suggest that ivermectin inhibits the growth of esophageal cancer both *in vitro* and *in vivo*. It had been reported that ivermectin inhibited breast and ovarian cancer cells proliferation by promoting PAK1 ubiquitination degradation and cytostatic autophagy by suppressing Akt/mTOR signaling pathway [13, 14]. The anti-proliferative effect of ivermectin on glioma cells was implicated with ivermectin-indcued cell cycle arrest and apoptosis [23]. Here, our results on ESCC cells indicated that the anti-proliferative effect of ivermectin on ESCC was significantly mediated via apoptosis. We found that treatment with ivermectin significantly increased nuclei fragmentation, apoptosis and G1 cycle arrest in ESCC cells.

NF-κB signaling pathway has obvious functions of inhibiting apoptosis, promoting cell proliferation and immune activation, and is also closely related to the differentiation, invasion and migration of several tumor cells [25]. Previous study had demonstrated that NF-κB activation promoted transcription of Bcl-2, VEGF, MMP and inhibited Bax expression, which reduced apoptosis and contributed to angiogenesis and progression of ESCC [26]. Blocking of NF-κB signaling pathway significantly suppressed ESCC cells growth, prevented angiogenesis and metastasis of ESCC, and sensitized ESCC to chemotherapeutic drugs [27, 28]. We also found that NF-κB pathway was markedly suppressed after treatment with ivermectin. Consistent with previous research, our results indicated ivermectin inhibited the growth of ESCC cells by regulating the NF-κB signaling pathway-mediated apoptosis, at least in part, mediated by regulation of Bax and Bcl-2 expression. The above results show that ivermectin, as an external stimulus signal, blocks further activation of the NF-κB signaling pathway, thereby attenuating the anti-apoptotic effect, and the downstream apoptotic signal further promotes apoptosis.

Mitochondrion is the central of cytosolic signaling transduction and plays a vital role in apoptotic pathway and is implicated in several anticancer drugs [22, 29, 30]. Recently research determined that ivermectin suppresses tumour growth and metastasis through degradation of PAK1 in ESCC [31]. However, how the mitochondria is involved in ivermectin-induced apoptosis remains unclear. Here, we determined mitochondrial dysfunction in ESCC cells treated with ivermectin, seen in the reduced mitochondrial DNA contents, decreased mitochondrial membrane potential and inhibited ATP production. Ivermectin markedly increased ROS levels of ESCC cells and scavenging the ROS by NAC surprisingly blocked the apoptosis and NF-κB inactivation, suggesting that ROS-mediated apoptosis was the main mechanism for ivermectin activity, while other pathways may also play a role to be explored in the future. The results indicated that mitochondrial dysfunction-derived ROS maybe trigger NF-κB signaling pathway and induce apoptosis by ivermectin treatment.

## Conclusions

We demonstrated that ivermectin effectively inhibit the proliferation of ESCC cells by inducing mitochondrial dysfunction, suppressing NF-κB signaling and promoting apoptosis. Our results suggest that ivermectin may be a potential therapeutic target against ESCC.

## Supplementary Information

**Additional file 1.** The original blots to related Fig. 2. A. The original blots to related Fig. 2A; B. The original blots to related Fig. 2C.

**Additional file 2.** The original blots to related Fig. 3. A. The original blots to related Fig. 3B; B. The original blots to related Fig. 3C.

**Additional file 3.** The original blots to related Fig. 4. A. The original blots to related Fig. 4A; B. The original blots to related Fig. 4C.

**Additional file 4.**

## Data Availability

The datasets used in the current study are available from the corresponding author on reasonable request.

## Abbreviations

ESCC: Esophageal squamous cell carcinoma
LDH: Proliferation and lactate dehydrogenase
EdU: Ethynyl deoxyuridine
MMP: Mitochondrial membrane potentialCellular
ROS: Reactive oxygen species
IC50: The half maximal-inhibitory concentration
NAC: Free radical scavenger N-acetyl-L-cysteine
