# Supplementary material for: Ivermectin induces apoptosis of esophageal squamous cell carcinoma via mitochondrial pathway
Source: BMC Cancer. 2021 Dec 7;21:1307. doi: 10.1186/s12885-021-09021-x (PMC8650430; doi:10.1186/s12885-021-09021-x)
Supplement: Supplementary file 1 — Additional file 1. The original blots to related Fig. 2. A. The original blots to related Fig. 2A; B. The original blots to related Fig. 2C. [file 12885_2021_9021_MOESM1_ESM.docx]

**
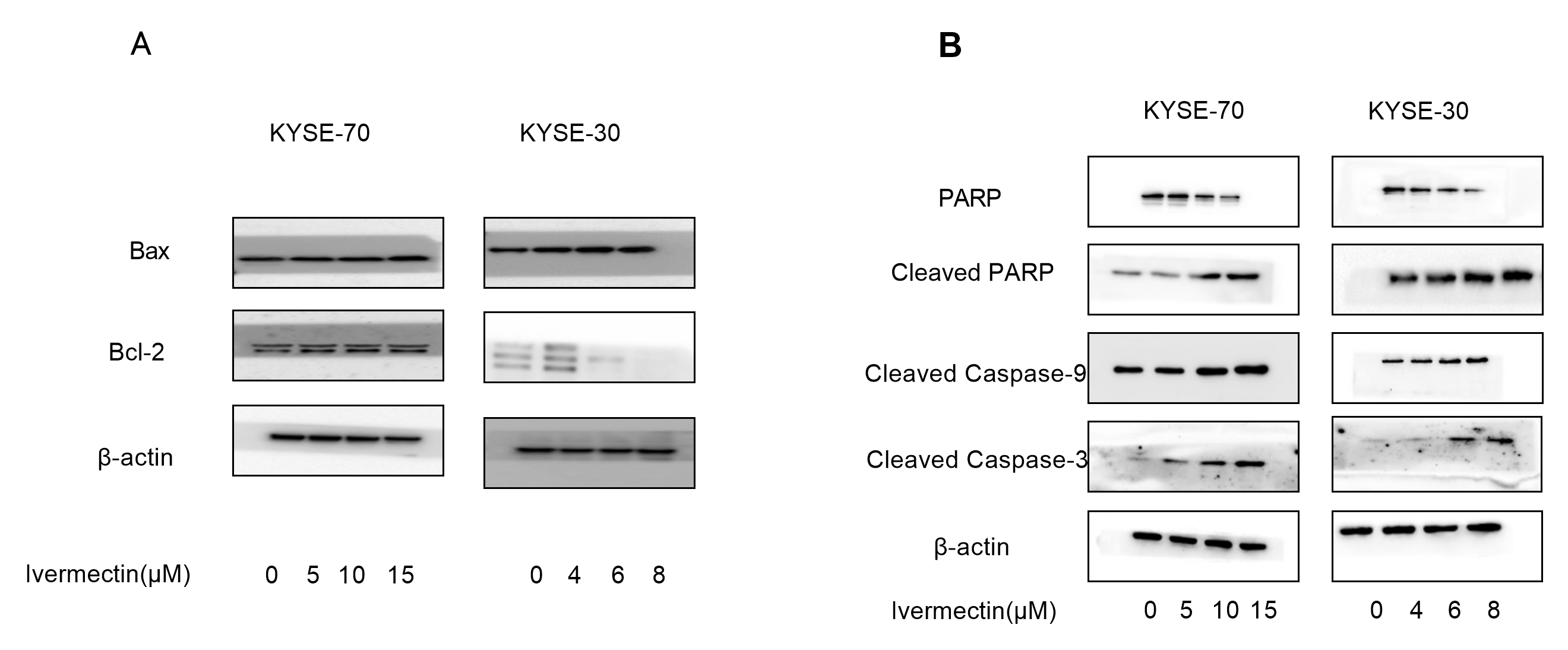
**

**Additional file 1** The original blots to related Fig. 2. A. The original blots to related Fig. 2A; B. The original blots to related Fig. 2C.
