# Supplementary material for: Ivermectin induces apoptosis of esophageal squamous cell carcinoma via mitochondrial pathway
Source: BMC Cancer. 2021 Dec 7;21:1307. doi: 10.1186/s12885-021-09021-x (PMC8650430; doi:10.1186/s12885-021-09021-x)
Supplement: Supplementary file 2 — Additional file 2. The original blots to related Fig. 3. A. The original blots to related Fig. 3B; B. The original blots to related Fig. 3C. [file 12885_2021_9021_MOESM2_ESM.docx]

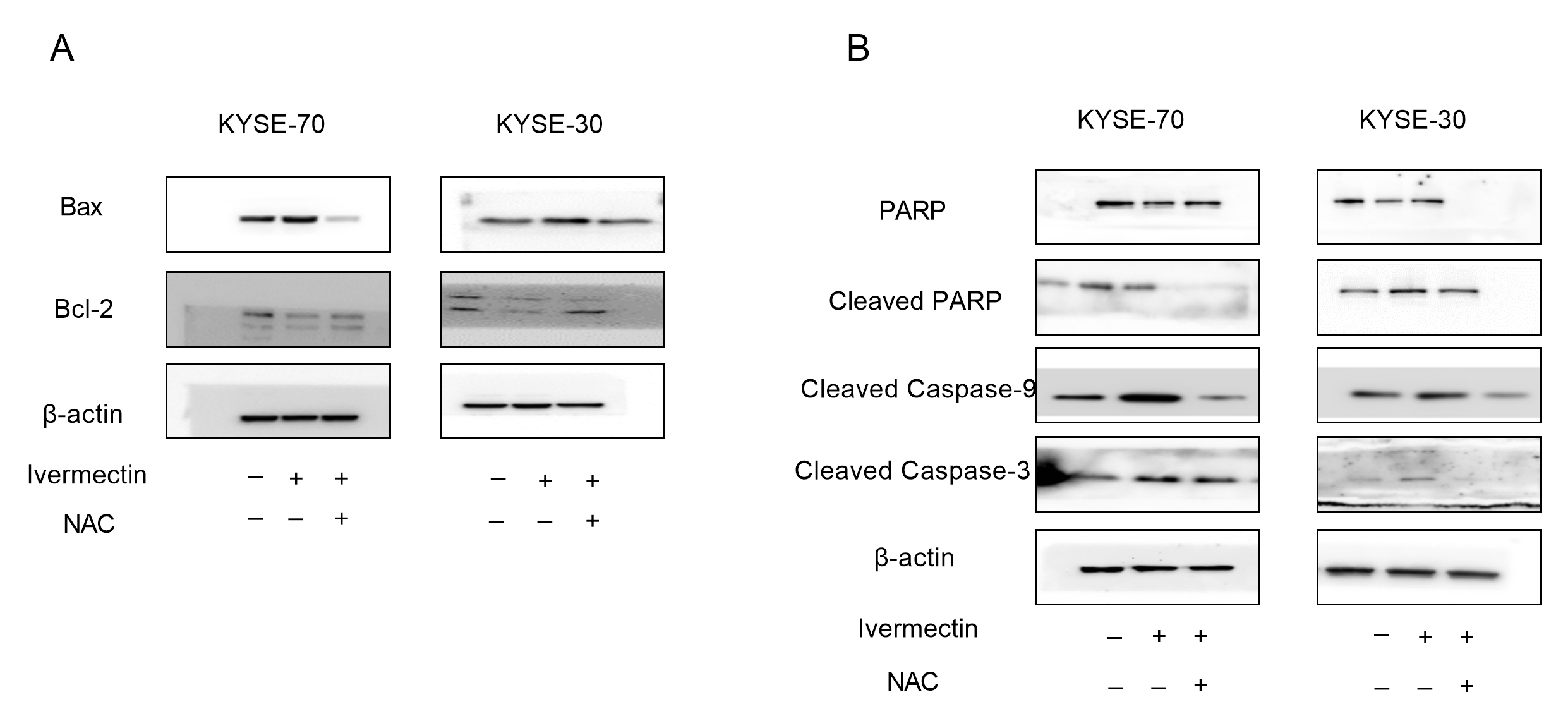


**Additional file 2** The original blots to related Fig. 3. A. The original blots to related Fig. 3B; B. The original blots to related Fig. 3C.
