# Supplementary material for: Ivermectin induces apoptosis of esophageal squamous cell carcinoma via mitochondrial pathway
Source: BMC Cancer. 2021 Dec 7;21:1307. doi: 10.1186/s12885-021-09021-x (PMC8650430; doi:10.1186/s12885-021-09021-x)
Supplement: Supplementary file 4 — Additional file 4. [file 12885_2021_9021_MOESM4_ESM.docx]

**Supplementary Table 1. Reagents**

| Reagent or resource | Source | Identified | Clone number | Working dilutions |
| --- | --- | --- | --- | --- |
| Bcl-2 Antibody | Proteintech | Cat#12789-1-AP | Polyclonal Antibody | WB: 1/1000 |
| Bax Antibody | Proteintech | Cat# 50599-2-lg | Polyclonal Antibody | WB: 1/5000 |
| Beta Actin Antibody | Proteintech | Cat# HRP-60008 | 7D2C10 | WB: 1/10000 |
| NF-κB P65 (D14E12) XP Rabbit mAb | Cell Signaling | Cat# 8242T | D14E12 | WB: 1/1000  IHC:1/1000 |
| Phospho-NF-κB p65 (Ser536) (93H1) Rabbit mAb | Cell Signaling | Cat# 3033T | 93H1 | WB: 1/1000  IHC:1/1000 |
| IκBα (L35A5) Mouse mAb | Cell Signaling | Cat# 4814T | L35A5 | WB: 1/1000 |
| Phospho-IκBα（Ser32)(14D4) Rabbit mAb | Cell Signaling | Cat# 2859T | 14D4 | WB: 1/1000 |
| Anti-PARP-1 Antibody | Santa Cruz Biotechnology | Cat# sc-56197 | 5A5 | WB: 1/1000 |
| Anti-cleaved PARP-1 Antibody | Santa Cruz Biotechnology | Cat# sc-56196 | 194C1439 | WB: 1/1000 |
| Cleaved Caspase-3 Antibody | Abways | Cat# CY5031 | No Applicable | WB: 1/2000  IHC: 1/100 |
| Cleaved Caspase-9 Antibody | Abways | Cat# CY5682 | No Applicable | WB: 1/2000 |
| Ki67 Antibody | Abways | Cat# CY5542 | No Applicable | IHC: 1/100 |
| ROS1 | Abcam | Cat# ab189925 | EPMGHR2 | IHC: 1/200 |
| Goat anti-Mouse IgG (H+L),HRP | Proteintech | Cat# SA00001-1 | Polyclonal Antibody | WB: 1/5000 |
| Goat anti-Rabbit IgG (H+L),HRP | Proteintech | Cat# SA00001-2 | Polyclonal Antibody | WB: 1/5000 |
| NAC | Sigma-Aldrich | Cat# A7250 |  |  |
| Pentobarbital | Sigma-Aldrich | Cat# P3761 |  |  |
| Hoechst 33342 | UE | Cat# H4047 |  |  |
| Immunohistochemistry Kit | Sangon Biotech | Cat# D601037 |  |  |
| Cell Cycle and Apoptosis Kit | US Everbright | Cat# C6031 |  |  |
| Ivermectin | Selleck Chemicals | Cat# S1351 |  |  |
| Reactive Oxygen Species Assay Kit | Beyotime | Cat# S0033S |  |  |
| TUNEL Assay Kit | UE | Cat# T6013 |  |  |
| Mitochondrial membrane potential assay kit with JC-1 | Beyotime | Cat# C2006 |  |  |
| MTT | Solarbio | Cat# M8180 |  |  |
| LDH assay kit | Beyotime | Cat# C0017 |  |  |
| ATP assay kit | Promega | Cat# FF2000 |  |  |
| EdU incorporation assay Kit | Ribobio | Cat# C10310 |  |  |

**Supplementary Table 2. Primer sequences**

| Primer name | Primer sequences (5’-3’) |
| --- | --- |
| Bax-F | GACGAACTGGACAGTAACATG |
| Bax-R | AGGCACCCAGGGTGATGCAA |
| Bcl-2-F | GTGGAGGAGCTCTTCAGGGA |
| Bcl-2-R | AGGCACCCAGGGTGATGCAA |
| GAPDH-F | ACGGATTTGGTCGTATTGGG |
| GAPDH-R | TGATTTTGGAGGGATCTCGC |
| ND1-F | CCCTAAAACCCGCCACATCT |
| ND1-R | GAGCGATGGTGAGAGCTAAGGT |
| 36B4-F | CAGCAAGTGGGAAGGTGTAATCC |
| 36B4-R | CCCATTCTATCATCAACGGGTACAA |
